# Committee on Litter

**Published:** 1895-03

**Authors:** 


TO MILITARY AND NAVAL MEDICAL OFFICERS.—The undersigned members of the committee on litter appointed by the Association of Military Surgeons of the United States to report at the approaching annual session, (Buffalo, N. Y., May 21, 22 and 23, 1895,) a desirable form of military litter for the comfortable, safe and expeditious transportation of the sick and wounded, solicit from medical officers of the national services and the national guard of the several states suggestions, plans or models of such an appliance to be delivered to either of them at their respective addresses on or before the 1st of April, 1895. Albert L. Gihon, medical director, United States Navy, United States Naval Hospital, Washington, D. C.; John Van R. Hoff, major and surgeon, United States Army, Governor's Island, Harbor of New York ; Myles Standish, captain and assistant surgeon, M. V. M., No. 200 Dartmouth street, Boston, Mass.

